# Effect of Non-Steroidal Anti-Inflammatory Drugs on Bone Healing 

**DOI:** 10.3390/ph3051668

**Published:** 2010-05-25

**Authors:** Jessica Cottrell, J. Patrick O’Connor

**Affiliations:** Department of Biochemistry & Molecular Biology, University of Medicine and Dentistry of New Jersey—New Jersey Medical School, Newark, NJ 07013, USA

**Keywords:** NSAIDs, bone healing, COX-2, COX-1, fracture

## Abstract

Nonspecific and COX-2 selective nonsteroidal anti-inflammatory drugs (NSAIDs) function by inhibiting the cyclooxygenase isoenzymes and effectively reduce pain and inflammation attributed to acute or chronic musculoskeletal pathologies. However, use of NSAIDs as an analgesic is thought to negatively contribute to bone healing. This review strived to provide a thorough unbiased analysis of the current research conducted on animals and humans regarding NSAIDs and their effect on bone healing. Specifically, this review discusses the role of animal models, dosing regiments, and outcome parameters when examining discrepancies about NSAIDS and their effects on bone regeneration. The role of COX-2 in bone regeneration needs to be better defined in order to further elucidate the impact of NSAIDs on bone healing.

## 1. Prostaglandins, Cyclooxygenases, and Bone Metabolism

Vertebrate bone is in a constant state of flux between destruction of old tissue and synthesis of new in a process often referred to as bone remodeling. An imbalance in bone remodeling can lead to osteoporosis or osteopetrosis [1]. Prostaglandins synthesized from arachidonic acid via cyclooxygenase activity mediate bone destruction and bone formation. Cyclooxygenase (COX) is the initial enzymatic activity in the conversion of arachidonic acid into prostaglandins. Two distinct forms of cyclooxygenase have been isolated, COX-1 and COX-2. COX-1 is constitutively expressed and is involved in physiological functions such as gastric protection and hemostasis, while COX-2 is inductively expressed and is involved in pathophysiological processes such as pain, inflammation, and fever. While the molecular and physiological connections that might explain this paradox of prostaglandin induced bone destruction and formation are not well understood, the basis of the paradox lies in the fact that prostaglandins can independently stimulate osteoclast and osteoblast activity to, respectively, destroy and synthesize bone [2]. Given the clear-cut effects of prostaglandin metabolism on normal bone physiology, the question arises as to what role cyclooxygenase metabolism and non-steroidal anti-inflammatory drugs (NSAIDs) which inhibit cyclooxygenase activity have during bone healing when new bone is rapidly made and remodeled into mature lamellar bone.

**Figure 1:**
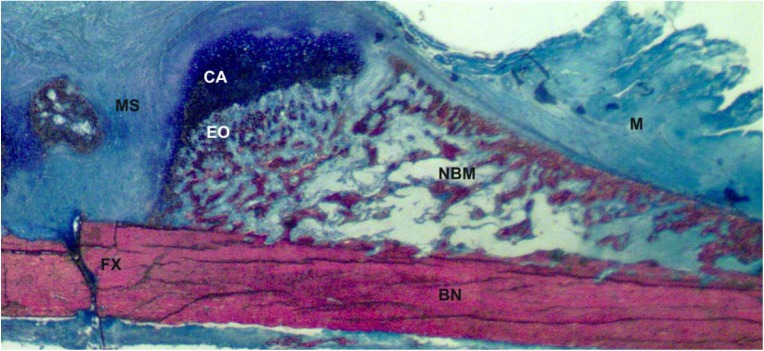
Upper right quadrant of a longitudinal rat fracture femur section, 2-weeks post-fracture. MS = mesenchymal cells, CA = cartilage, EO = site of endochondral ossification, FX = fracture site, BN = bone, M = muscle, NBM = site of new bone and marrow.

## 2. Types of Bone Healing

Unlike most mammalian tissues, bone heals by a regenerative process to restore the metabolic and mechanical functions of the injured bone. Bone is a highly perfused tissue so that following a typical bone fracture localized tissue hypoxia and hematoma formation occurs [3]. Inflammation at the fracture site soon follows and is characterized by cellular infiltration with large numbers of T-cells, granulocytes, and CD68-positive mononuclear cells and a paucity of B-lymphocytes [4,5]. It is thought that cell signaling events associated with hypoxia, degranulation of platelets during hematoma formation, or inflammatory cells initiate the bone healing pathway. The fracture site is subsequently invaded with a large number of fibroblast-like cells as the fracture callus forms (Figure 1). The exact origin of these fracture callus cells is unknown but it is likely that the fracture callus cells have multiple origins including proliferating periosteal and endosteal cells, circulating mesenchymal stem cells, pericytes, and muscle-derived mesenchymal stem cells. At each periphery of the fracture callus, a small amount of new bone is formed via intramembraneous ossification by osteoblasts in the periosteum [6]. These sites of new bone act as buttresses for the fracture callus and aid in the initial mechanical stabilization of the fracture. Fracture callus cells adjacent to the newly formed bone and the periosteum differentiate into chondrocytes such that the early fracture callus has new bone at its peripheries, fibroblast-like cells in the middle, and chondrocytes sandwiched in between. The chondrocytes closest to the periosteum and new bone are the first chondrocytes to become hypertrophic and form calcified cartilage. Osteoblast proliferating from the pre-existing bone beneath the calcified cartilage, and in conjunction with angiogenic activity, form bone on the calcified cartilage stratum. This bone formation process is endochondral ossification. As healing progresses, differentiation of the fracture callus cells into chondrocytes proceeds from the periphery of the callus and the original periosteum towards the center and circumferential edges of the callus. Behind the newly differentiating chondrocytes, calcified cartilage formation and endochondral ossification occur until the fracture gap is bridged with newly formed bone. Subsequent bone remodeling enhances the mechanical properties of the newly formed bone tissue and returns the bone into its former shape. This is the normal healing response to a broken bone and is often referred to as secondary healing. 

Primary bone fracture healing relies upon the constant and natural remodeling processes of the skeleton to heal a fracture. It is generally not a natural healing event but is the basis of many orthopaedic surgical procedures. In this scenario, the two bone ends of the fracture are juxtaposed into their original configuration and then locked in place by surgically applied plates, rods, pins, screws, or some combination [7]. This causes rigid fixation of the fracture and the natural process of bone remodeling knits the fractured bone ends together. Primary healing is probably spurred by the initial hypoxia, hematoma, and inflammation reactions that occur prior to surgery and the stimulation of remodeling that occurs due to osteonecrosis of the bone ends caused by the hypoxia.　 

Spinal fusion is an artificial type of bone healing that is used to fuse two or more vertebrae often for arthritic pain relief. For instance, a posterolateral spinal fusion involves dissecting the soft tissues from the dorsal side of the vertebrae and abrading the bone surface of the vertebrae to stimulate periosteal osteogenesis [8]. Bone autograft, generally harvested from the hip, is morselized and packed between the two vertebrae to be fused. The vertebrae are stabilized with various fixation devices that are applied to the dorsal-lateral or ventral-lateral aspects of the vertebrae. The abraded bone surfaces and the autograft bone chips begin forming new bone primarily through an intramembraneous ossification process. However, small islands of cartilage are often observed in the fusion mass indicating that unorganized endochondral ossification also occurs during spinal fusion to form new bone. The ultimate goal is that the new bone formed from the vertebral surfaces and the autograft unite and remodel into a solid bone mass thereby stopping or dramatically reducing movement between the two vertebrae.

Small bone defects, such as burr holes, heal by direct synthesis of new bone to fill the defect. This probably occurs in conjunction with the normal processes of bone remodeling. Similarly, other types of arthrodesis procedures to fuse two or more bones or bone growth into or around implanted devices, such as intramedullary stems for artificial hips, likely relies upon direct bone synthesis and not endochondral ossification that occurs during fracture healing. Thus, NSAID effects on fracture healing may not directly relate to other, specific bone formation processes.

## 3. Cyclooxygenase Inhibitors

Nonsteroidal anti-inflammatory drugs are commonly prescribed to alleviate acute and chronic musculoskeletal pain. Acute musculoskeletal injuries are characterized by localized tissue swelling, inflammation, and pain and commonly include abnormally stretched or partially torn ligaments and tendons or bone fractures. Chronic musculoskeletal pathologies such as osteoarthritis or Paget's disease are also associated with localized inflammation and tissue swelling. Treatment with NSAIDs reduces local inflammation and pain by inhibiting cyclooxygenase, which is the rate-limiting enzymatic activity in the conversion of arachidonic acid to pro-inflammatory prostaglandins. As a result, NSAID inhibition of cyclooxygenase reduces prostaglandin levels, which impedes inflammation and the attending swelling and pain.

Traditional NSAIDs inhibit the cyclooxygenase activity of both cyclooxygenase-1 (COX-1) and cyclooxygenase-2 (COX-2) with near equal potency and can induce many side effects. NSAID use can affect blood pressure, ovulation, kidney function, and most frequently the gastrointestinal system [9]. In fact, NSAID use can cause gastrointestinal bleeding and perforations that can be lethal. NSAID side effects were attributed to inhibition of COX-1 because it is constitutively expressed and functions in the protection of gastric mucosa and regulation of platelet aggregation [10,11].　　 

In 1989, a second inducible form of the cyclooxygenase enzyme, cyclooxygenase-2 (COX-2) was discovered [12,13,14]. This discovery spurred interest in defining the connection between cyclooxygenase activity and inflammation in order to understand NSAID use and its side effects. COX-2 expression was found to be induced by tissue injury, certain cell-signaling events, or after noxious stimuli associated with inflammation [15,16,17]. Soon after, NSAID therapy was hypothesized to reduce pain and inflammation by primarily inhibiting COX-2 and not COX-1. As a result, new drugs that selectively inhibit COX-2 were developed. These COX-2 selective inhibitors were hypothesized to reduce gastrointestinal and other side effects because the homeostatic functions of COX-1 would be unaffected. In 1999, celecoxib and rofecoxib became the first COX-2 selective inhibitor drugs available for clinical use. Early clinical trials showed an increased risk for cardiovascular effects caused by rofecoxib, and in 2004 this product was withdrawn from the market, while celecoxib is still available. Celecoxib is still one of the most commonly prescribed drugs in the United States because it has reduced effects on gastric mucosa [18] although it still poses a cardiovascular risk as do other NSAIDs [19].

Because NSAIDs are commonly used to treat skeletal injuries and pain, their effects on fracture healing have been examined by many investigators over the past 35 years [20,21,22,23,24,25,26,27,28]. These studies have shown that the administration of NSAIDs and COX-2 selective NSAIDs can impair or delay bone healing and decrease the mechanical integrity of the healing bone.

## 4. NSAID Effects on Experimental Models of Bone Healing

### 4.1. Effects of Traditional NSAIDs on Bone Healing

Many studies have shown that traditional NSAIDS (nonspecific) such as aspirin, indomethacin, and ibuprofen (our definition of NSAID does not include acetaminophen) impede bone healing in various animal models (see Table 1) [20,29,30,31,32,33,34,35,36,37,38,39,40,41,42,43,44,45,46,47,48]. 

**Table 1:** NSAID effects on bone healing: animal studies.

NSAID	Animal; Sex, Age, Weight	Bone Healing Model	Dose(s) mg/kg/day	Drug Administration (Post-Procedure)	Assay(s)	Longest Time Point	Outcome	Comments	Ref.
Aspirin	Rat, Male, 45 days	Closed, Unstable, Radius and ulna fractures	100, 200, 300	PO for 21 days	Histology	22 days	Inhibition at highest dose		[23]
Aspirin	Rabbit, N/A	Bone ingrowth, femur	17, 34	SQ injection	Histology	8 weeks	Inhibition at high dose	Porous-coated chrome-cobalt implants; 4.5 mm diameter, 7 mm length	[38]
Celecoxib	Mice, 8–10 wks, ≈25 g	Closed, Stable, Tibia fracture	10, 50	In mice chow as a peanut butter pellet, PO daily till endpoint	Histology, mechanics	12 weeks	No effect		[52]
Celecoxib	Rat, Male, 6–9months, 584±62 g	Closed, Stable, Femur fracture	4	PO daily till endpoint	Histology, Radiography, Mechanics	8 weeks	Inhibition		[25]
Celecoxib	Rat, Male, 300 g	Closed, Stable, Femur fracture	3	Diet daily till endpoint	Radiography, histology mechanics	12 weeks	No effect	Drug was given in chocolate	[51]
Celecoxib	Rat, Female, 281±20 g	Closed, Stable, Femur fracture	3, 6	PO daily till endpoint	Radiography, histology, mechanics	8 weeks	Inhibition		[73]
Celecoxib	Rat, Female, 272±7 g	Closed, Stable, Femur fracture	2 , 4, 8	5-day before PO, PO daily till endpoint, 7 to 28 days daily, 14 to 28 days daily	Radiography, mechanics	8 weeks	Inhibition, all doses, over time course, pre 5 day dose had no effect		[64]
Celecoxib	Rat, Female, 250–300 g	Closed, Stable, Femur fracture	4	PO BID daily till endpoint	Radiography, histology, mechanics	5 weeks	Inhibition		[98]
Celecoxib	Rabbit, Male, 4.3–5.4 kg	Spinal fusion	10	PO daily till endpoint	Radiography, histology	8 weeks	No effect		[41]
Diclofenac	Rat, Male, 4–8 months 220–300 g	Closed, Stable, Tibia fracture	1, 2	PO daily for 10 days	Radiography, histology	6 weeks	No effect		[60]
Diclofenac	Rat, Male, 300–350 g	Open, Stable, Tibia fracture	5	PO daily for 7 or 21 days	Radiography, mechanics, CT scan	3 weeks	Inhibition		[42]
Diclofenac	Rat, Male, 30–350 g	Open, Stable, Tibia Osteotomy	5	PO daily for 7 days or 21 days	Histology	3 weeks	3 week dose inhibited callus maturation		[48]
Etodolac	Rat, Female, 250–300 g	Closed, Stable, Femur fracture	20	PO daily till endpoint	Radiography, Mechanics	3 weeks	Inhibition		[24]
Etodolac	Rat, 12 wks, 250–300 g	Closed, Stable, Femur fracture	20	PO daily for 1 week, 3 weeks or during week 3 only	Radiography, mechanics	3 weeks	Inhibition, when administered for 1 week or 3 weeks		[26]
Flunixin	Rabbit, Female, 2.6–3.0 kg	Closed, Unstable, Tibia fracture	1.1	PO daily till endpoint	Mechanics,	3 weeks	No effect		[58]
Ibuprofen	Mice, Male, 8–10 wks, ≈25 g	Closed, Stable, Tibia fracture	30	In mice chow as a peanut butter pellet, PO daily till endpoint	Histology, mechanics	12 weeks	No effect		[52]
Ibuprofen	Rat, Male, 430–530 g	Closed, Unstable, Tibia fracture	30–35	Beginning 1 week prior to surgery and PO for 5 days a week	Callus size, calcium activity	9 weeks	Ibuprofen activates calcium metabolism & decreases bone mass and composition		[31]
Ibuprofen	Rat, Male, 430–530 g	Closed, Unstable, Tibia fracture	30–35	Beginning 1 week prior to surgery and PO for 5 days a week	Histology, mechanics	9 weeks	Inhibition		[32]

Ibuprofen	Rat, Male, 440–500 g	Closed, Stable, Tibia fracture	30	PO for 5 days/week, beginning 3 days post-fracture till endpoint	Histology, mechanics	12 weeks	No effect		[59]
Ibuprofen	Rat, Female, 375–450 g	Closed, Stable, Femur fracture	30	Diet for 4 or 12 weeks	Histology, mechanics	12 weeks	Inhibition		[49]
Ibuprofen	Rat, Male, 300 g	Closed, Stable, Femur fracture	30	PO daily till endpoint	Radiography,	4 weeks	Inhibition		[45]
Ibuprofen	Rabbit, Male & Female, 4 months, 2.1–3.5 kg	Open, Unstable, Femur osteotomy	7.5	PO daily till endpoint	Mechanics	8 weeks	Inhibition		[34]
Ibuprofen	Rabbit, N/A	Bone ingrowth, femur	17, 34	SQ injections	Histology	8 weeks	Inhibition, both doses, with dose response	Porous-coated chrome-cobalt implants; 4.5 mm diameter, 7 mm length	[38]
Ibuprofen	Rabbit, Male, 3.5 kg	Open, Fibula, Osteoto my	50	PO three times daily for 28 days	Histology, mechanics	12 weeks	Inhibition		[28]
Indomethacin	Mice, Male, 8–10 wks, ≈25 g	Closed, Stable, Tibia fracture	2	In mice chow as a peanut butter pellet, PO daily till endpoint	Mechanics	12 weeks	No effect		[52]
Indomethacin	Rat, Male, 50 days, ≈160 g	Tooth extraction	4	PO, BID 2mg/kg/day for 5 days	Histology	3 weeks	Inhibition	NSAID treated rats had delayed healing (1 week delay)	[29]
Indomethacin	Rat, Male, Adolesce nt	Closed, Unstable, Femur fracture	2	PO daily till endpoint	Radiography, histology, mechanics	24 days	Inhibition		[20]
	
Indomethacin	Rat, Male, 195±5 g	Closed, Unstable, Femur fracture	2	PO daily till endpoint	Callus weight, histology	12 days	Indomethacin does not affect collagen metabolism		[55]
Indomethacin	Rat, Male, 210–295 g	Closed, Unstable, Femur fracture	2	PO daily till endpoint	Radiography, manual assessment	94 days	Inhibition		[22]
Indomethacin	Rat, Male, 45 days	Closed, Unstable,	1, 2, 4	PO daily for 21 days	Histology	22 days	Inhibition at all doses		[23]
Indomethacin	Rat, Male, 2, 6–7, or 8–9 months	Fracture by drill hole in caudal vertebra	4	Beginning 1 week prior to surgery & daily till endpoint	Histology	56 days	Inhibited	No effect if Rx was stopped day after lesion was induced	[33]
Indomethacin	Rat, Male, 4 wks	Drill hole in calvaria, 0.8mm	2	PO daily till 1, 2 or 4 weeks	Radiography, histology	4 weeks	Inhibition	Dexamethasone was also tested and was found to inhibit bone wound healing more	[35]
Indomethacin	Rat, Male, 315–355 g or 329–479 g	Open, Stable, Femur fracture	2	1 hr prior to surgery, PO daily for 3 days	Mechanics	6 weeks	Inhibition		[107]
Indomethacin	Rat, Male, 67–82 g or 52–64 g	Closed, Unstable, Femur fracture	0.5, 2	2 mg/kg dose was given PO daily for 10 days; A single dose of 0.5 mg/kg was injected at fracture site	Radiography,	20 days	Inhibition was found with oral and local treatment	Injection at fracture site was given in a poly-orthoester gel	[39]
Indomethacin	Rat, Female, 375–450 g	Closed, Stable, Femur fracture	1	Diet for 4 or 12 weeks	Histology, mechanics	12 weeks	Inhibition		[49]
Indomethacin	Rat, Male, 10–12 wks, 370–421 g	Spinal fusion	3	SQ injection, 6 days/week till endpoint	Manual assessment	12 weeks	Inhibition		[79]
Indomethacin	Rat, Female, 294±4 g	Mechanical loading	0.02, 0.2, 2	A single dose given 3 hours prior to loading	Histology	12 days	Partially inhibited		[53]
Indomethacin	Rat, Male, 335–345 g	Open, Stable, Femur osteotom y	2	IM for 3 days	Mechanics	6 weeks	Inhibition		[54]
Indomethacin	Rat, Male, 6–9 months, 584±62 g	Closed, Stable, Femur fracture	1	PO daily till endpoint	Radiography, histology, mechanics	8 weeks	Inhibition		[25]
	
Indomethacin	Rat, Male, ≈300 g	Closed, Stable, Femur fracture	1	PO daily till endpoint	Radiography, histology, mechanics	12 weeks	No effect by 12 weeks	Indo delayed at 4 wks mechanically	[51]
Indomethacin	Rat, Female, ≈226 g	Closed, Stable, Tibia fracture	0.625	IP, prior to surgery, BID for 7 days	Radiography, histology, mechanics	3 weeks	Inhibition		[50]
Indomethacin	Rabbit, 4.5 months, 2.0–2.7 kg	Open, Unstable, Radius & ulna osteotomy	10, 5	Fed daily, 6 days a week; high dose for the first 2 weeks and then low dose for next 4 weeks	Radiography, histology	43 days	Inhibition		[30]
	
Indomethacin	Rabbit, Male & Female, 4 months, 2.1–3.5 kg	Open, Unstable, Femur osteotomy	5	PO daily till endpoint	Mechanics	8 weeks	Inhibition		[34]
Indomethacin	Rabbit, 4.3–6.0 kg	Open, Unstable, Tibia osteotomy	50	In drinking water, 4 days prior to surgery, PO daily till endpoint	Mechanics, bone mineral content	6 weeks	Inhibition		[36]
Indomethacin	Rabbit, Female, Juvenile	Open, Unstable, Femur osteotomy	10	SQ injections,	Radiography,	6 weeks	Uncertain effect		[56]
Indomethacin	Rabbit, N/A	Bone ingrowth, femur	1, 2, 3	SQ injections, daily	Histology	8 weeks	Inhibition, all doses, with dose response	Porous-coated chrome-cobalt implants; 4.5 mm diameter, 7 mm length	[38]
Indomethacin	Rabbit, Male	Bone ingrowth, femur	10	SQ injections, daily	Histology	8 weeks	Inhibition	Radially drilled, cylindrical implants	[37]
Indomethacin	Rabbit, 3.5 kg	Open, Unstable, Tibia osteotomy	12.5	In drinking water, 4 days prior to surgery, PO daily till endpoint	Histology	6 weeks	No effect		[61]
Indomethacin	Rabbit, Male, 4.3–5.4 kg	Spinal fusion	10	PO daily till endpoint	Radiography, histology	8 weeks	Inhibition		[41]
Indomethacin	Rabbit, 5 kg	Spinal fusion	10	Started 2 or 4 weeks after PO, daily till endpoint	Manual assessment	6 weeks	Inhibition when treatment was started at 2 weeks PO		[44]
Indomethacin	Rabbit, Male, 3 months, 3.5 kg	Open, Unstable, Ulna fracture	2	PO daily till endpoint	Histology, mechanics	6 weeks	Inhibition		[46]
Indomethacin	Dog, 12–21 kg	Open, Stable, Transsecti on of the 3rd metacarpu s	5	PO BID daily for 8 days	Radiography,	8 weeks	No effect		[57]
Ketorolac	Mice, Male, 8–10 wks. ≈25 g	Closed, Stable, Tibia fracture	2	In mice chow as a peanut butter pellet, length not mentioned	Histology, mechanics	12 weeks	Inhibition at 4 weeks		[52]
Ketorolac	Rat, Male, 335–345 g	Open, Stable, Femur osteotomy	1	IM for 3 days	Mechanics	6 weeks	Inhibition		[54]
Ketorolac	Rat, Male, 425–600 g	Closed, Stable, Femur fracture	4	PO daily till endpoint	Histology, mechanics, gene expression	35 days	Inhibition		[72]
Ketorolac	Rabbit, Male, 3.0 kg	2 cm defect, Ulna	2, 4	PO daily till endpoint	Radiography, histology	6 weeks	No effect with low dose, high dose inhibition detected between 2nd & 4th week	DBM was added in conjunction to ketorolac	[76]
Ketorolac	Rabbit, 4.0–4.5 kg	Spinal fusion	4	Continuous infusion (sq pump) for 7 days	Palpation	6 weeks	Inhibition	75%, 35%, and 100% fusion in the saline, ketorolac, and ketorolac plus BMP-2 groups, respectively	[71]
Meloxicam	Rabbit, Male, 3 month 3.5 kg	Open, Unstable, Ulna osteotomy	0.3	PO daily till endpoint	Histology, mechanics	6 weeks	Inhibition		[46]
Naproxen	Rabbit, Male, 6–12 months, 3.5–4.2 kg	Bone-ingrowth chamber	110	Water for 4 weeks	Histology	4 weeks	Inhibition	Proximal tibia site	[40]
NS-398	Rat, Female, 294±4 g	Mechanical loading	0.02, 0.2, 2	A single dose given 3 hours prior to loading	Histology	12 days	Inhibition		[53]
Parecoxib	Rat, Male, 425–600 g	Closed, Stable, Femur fracture	0.3, 1.5	PO daily till endpoint	Histology, mechanics, gene expression	35 days	Inhibition		[72]
Parecoxib	Rat, Female, ≈226 g	Closed, Stable, Tibia fracture	0.5	IP, prior to surgery, BID for 7 days	Radiography, histology, mechanics	3 weeks	Inhibition		[50]
Piroxicam	Rabbit, Female, 2.6–3.0 kg	Closed, Unstable, Tibia fracture	0.1, 0.2	PO daily till endpoint	Mechanics,	3 weeks	No effect		[58]
Rofecoxib	Mice , Male, 8–10wks. ≈25 g	Closed, Stable, Tibia fracture	1, 5	In mice chow as a peanut butter pellet, PO daily till endpoint	Histology, mechanics	12 weeks	Inhibition at 8 weeks		[52]
Rofecoxib	Mice, Male, 4 months	Open, Stable, Femur osteotomy	5	PO daily till endpoint	Radiography, histology, mechanics, laser doppler flow	32 days	Inhibition, affects blow flow across fracture gap		[75]
Rofecoxib	Rat, Male, 6–9 months, 584±62 g	Closed, Stable, Femur fracture	3	PO daily till endpoint	Radiography, histology, mechanics	8 weeks	Inhibition		[25]
Rofecoxib	Rat, Male, 300 g	Closed, Stable, Femur fracture	8	PO BID daily until endpoint	Radiography,	4 weeks	Inhibition		[45]
Rofecoxib	Rabbit, Male	Closed, Stable, Femur fracturer	3	PO daily for 4 weeks	Histology	4 weeks	Inhibition	Proximal tibia	[40]
Rofecoxib	Rabbit, Male, 6–12 months, 3.5–4.2 kg	Bone-ingrowth chamber	3	PO daily for 2 weeks, 6 weeks or last 2 weeks	Histology	6 weeks	Inhibition when administered for 6 weeks, no effect found when treatment was given for 2 weeks		[74]
Rofecoxib	Rabbit, Male, 3 months, 3.5 kg	Open, Unstable, Ulna fracture	0.5	PO daily till endpoint	Histology, mechanics	6 weeks	Inhibition		[46]
Rofecoxib	Rabbit, Male, 3.5 kg	Open, Fibula Osteotomy	50	PO 3x daily for 28 days	Histology,	12 weeks	Inhibition		[28]
Tenoxicam	Rat, Male, ≈100 g	Open, Unstable, Tibia Fracture	10	1 week prior to PO, PO or 48hrs after PO, than daily till endpoint, IM injections	Histology	4 weeks	Inhibition		[43]

* PO = post-op; SQ = subcutaneous; BID = twice a day, QID = three times a day; IP = intraperitoneal, IM = Intramuscular

In 1976, Rø *et al.* were the first to describe the effect of traditional NSAID use on fracture healing outcomes [20]. This study used a closed, non-stabilized femur fracture model in rats to demonstrate that indomethacin treatment delayed fracture healing. More specifically, indomethacin treatment slowed the resolution of the fracture hematoma, increased angulation between bone ends and reduced the biomechanical properties of the bone. Shortly after, Allen *et al.* supported these conclusions by demonstrating that indomethacin and aspirin caused drug- and dose-dependent delays in bone healing of rat radius and ulna fractures [23].

Research in the following decades continued to support the results of these earlier studies and strongly emphasized the negative effects of traditional NSAIDs on bone healing. For example, Altman *et al.* demonstrated that ibuprofen (30 mg/kg per day) and indomethacin (1 mg/kg per day) led to decreased mechanical properties and delayed maturation of the callus [49]. In a rat tibia fracture model, indomethacin treatment reduced bone mineral density (BMD) at the fracture site two weeks after fracture [50]. The reduced BMD correlated with decreased ultimate bending moment and bending stiffness at three weeks [27,50]. In 2002, Simon *et al.* showed that indomethacin treatment in a rat femur fracture model decreased callus mechanical properties at four and six weeks post-fracture. However, the biomechanical properties between control and indomethacin-treatment group fracture calluses were similar by eight weeks [25]. Other investigators have shown that the reduced bone strength associated with indomethacin treatment at earlier time points had dissipated by 12 weeks post-fracture [51,52]. These results demonstrate that the non-selective NSAIDs delay fracture healing but do not have any detrimental effects on the ultimate fracture healing outcome in these animal models. Most studies using osteotomy and bone ingrowth models have demonstrated that indomethacin and ibuprofen treatment retards bone healing [29,33,34,35,36,37,38,48,53,54]. In contrast, only a few studies indicate that NSAIDs have little or no effect on fracture healing outcomes [55,56,57,58,59,60]. One study concluded that indomethacin treatment had no effect on cortical bone healing following a small drill hole defect (2 mm in diameter) [61]. However, the scope of this study was limited to testing a single time point (six weeks) and one outcome parameter. Other studies have shown that non-selective NSAIDs delay fracture healing in larger animal models such as rabbits and dogs [21,28,30]. 

Bone resorption and formation can be regulated by prostaglandin E_2_ [62]. Prostaglandin E and F have also been shown to be released after fracture [63]. Data from Simon *et al.* demonstrates that treatment with nonselective or COX-2 specific NSAIDS at doses comparable to those given to humans (diclofenac, 5 mg/kg or celecoxib, 4 mg/kg) reduced fracture callus levels of prostaglandin E_2_ and F_2α_ while negatively affecting fracture healing [64]. The reduction of prostaglandins in the environment of the healing bone is thought to contribute to the poor healing of the bone.

Other common side effects of NSAID use include nephrotoxicity, delayed blood clotting, and gastrointestinal bleeding [49,65,66,67]. Chronic NSAID therapy or acute high doses of NSAIDs in animals and humans can cause perforations and gastrointestinal bleeding, which is sometimes lethal [68]. According to Lanas *et al.* mortalities related to NSAID use and gastrointestinal complication are estimated to be approximately 59 people per 1 million [69]. Though COX-2 selective NSAIDs were developed to avoid this complication and can reduce it, it appears that both COX-1 and COX-2 are necessary to prevent and heal gastrointestinal lesions, respectively [70].

### 4.2. Effects of COX-2 Selective Inhibitors on Bone Healing.

With the advent of COX-2 selective NSAIDs and their nominal advantages over traditional NSAIDs, physicians began to use them for acute and chronic pain management. As a result, researchers began to study the effects on COX-2 selective NSAIDs on bone healing. The effects of COX-2 inhibitors on bone healing are still highly debated. Many of the existing animal studies have found an inhibitory effect [25,40,46,52,53,64,71,72,73,74,75] but a few studies have found no lasting negative effects [41,51,52,76]. Major factors that may underlie the discrepancies between these studies are the variability in drug dosing, dosing duration, the number of animals used within each study, the age of the animal, the type of fracture model, experimental endpoints, and outcome measurements.

In 1996, Forwood *et al.* showed that NS-398, a COX-2 selective inhibitor, impaired mechanical loading induced bone formation [53]. More importantly, these results demonstrated that COX-2 was expressed in bone and had an important function. Simon *et al.* demonstrated that femur fracture healing was severely impaired in COX-2 null mice and in rats treated with celecoxib or rofecoxib; cementing the importance of COX-2 for bone healing [25]. X-ray and histological examination of femur fracture healing in COX-1 null and COX-2 null mice showed an abundant callus undergoing endochondral ossification in COX-1 null mice but an X-ray lucent, cartilaginous callus in the COX-2 null mice with little or no apparent endochondral ossification. Femur fracture healing in rofecoxib treated rats (3 mg/kg, QD) was also severely impaired based upon radiographic and histological observations and torsional mechanical testing. In contrast, celecoxib treatment (4 mg/kg, QD) appeared to be less deleterious to femur fracture healing in the rats based upon the torsional mechanical testing analysis [25]. Data from other researchers supported these results demonstrating that COX-2 inhibitors like celecoxib, rofecoxib, parecoxib, and meloxicam impair fracture healing in mice and rats [46,72,73,74,75]. For instance, Gerstenfeld *et al.* demonstrated that an oral dose of parecoxib (1.5 mg/kg) delayed femur fracture healing in male rats by decreasing material properties at three weeks after fracture and structural properties at five weeks after fracture [72].

In contrast, a few studies have shown that COX-2 inhibitors have no lasting effect on femur fracture healing [41,51,52]. Brown *et al.* examined the effects of celecoxib (3 mg/kg/day) on male rat femur fractures [51]. Drugs were administered daily in their food starting one day after fracture and continued until sacrifice. Healing was determined by qualitative histology, radiographic scoring, and mechanical testing at four, eight, and 12 weeks after fracture. The results demonstrated that celecoxib led only to early, negative histological changes, which showed no significant differences by 12 weeks. In a study, by Mullis *et al.* tibia fracture healing was measured in mice treated daily with peanut butter pellets of celecoxib (10 mg/kg or 50 mg/kg) and rofecoxib (1 mg/kg and 5 mg/kg) [52]. Healing was measured by histological analysis at two weeks and biomechanical testing at four, eight, and 12 weeks. Only the low dose rofecoxib (1 mg/kg) treatment led to decreased mechanical properties at eight weeks after fracture but by 12 weeks no difference was found.

To address these discrepancies, a comprehensive study was performed to determine the dose and time-dependent effects of COX-2 selective NSAID therapies on fracture healing in rats [64]. The previous studies that tested the effects of celecoxib on fracture healing used male rats in which celecoxib has an elimination time of approximately four hours [25,41,51,52,72,75]. In contrast, this study used female rats because the elimination time for celecoxib in female rats is approximately 11 hours which is very similar to that of humans [64]. The data from this study demonstrated that celecoxib given in the human therapeutic range (4 mg/kg, BID) during the early stages of fracture repair significantly reduced femur fracture callus mechanical properties at later stages of healing and increased the number of non-unions [64]. This study also demonstrated that delaying celecoxib treatment until seven days or more after fracture improved healing outcomes. Other studies support these results suggesting that avoiding NSAID treatment early during fracture healing may lessen its negative effects [72,78]. Overall, the study showed that the negative effects of celecoxib treatment on fracture healing were dependent upon drug dose, length of treatment, and when during fracture healing celecoxib treatment occurred. The study results indicate that the earlier, conflicting COX-2 selective NSAID animal studies likely reflect differences in the level and duration of COX-2 inhibition. Ultimately, the data support the conclusion that COX-2 selective NSAID use is detrimental to fracture healing.

### 4.3. Effects of NSAIDs on Spinal Fusion 　

Few studies exist which examine the role of NSAID treatment on spinal fusion outcomes in animal models. Most of these studies demonstrate that traditional NSAIDs inhibit spinal fusion success. For instance, Dimar *et al.* demonstrated that indomethacin treatment delayed healing in rats that underwent a three-level posterior spinal fusion [79]. In this study, 45% of control rat spines were completely fused while only 10% of the indomethacin treated rat spines were completely fused. In a rabbit spinal fusion study, only 18% of the rabbits given indomethacin orally after surgery had successfully fused vertebrae as compared to 64% in the control treatment group [41]. This same study also found that celecoxib treatment (10 mg/kg, daily for eight weeks) had no effect on spinal fusion success. A later rabbit study by the same group found similar negative effects caused by indomethacin treatment even when the treatment was delayed until two weeks after the spinal fusion surgery [44]. Research conducted using other traditional NSAIDs like ketorolac in the rabbit posterolateral spinal fusion model showed that its use decreased spinal fusion success from 75% in controls to 35% in the drug treatment groups [71]. A series of published reports from Scott S. Reuben concerning the use of COX-2 selective NSAIDs for spinal fusion surgery were found to be fraudulent and most have been retracted. As such, the published results of these reports are not discussed. Given the discrepancy between the celecoxib results and those from the indomethacin and ketorolac studies, additional studies are needed to define the effects of COX-2 selective inhibitors on spinal fusion.

## 5. Human Studies of NSAID Effects on Bone Healing and Formation

NSAID therapy can have a clinically significant effect on bone formation in humans. The negative effect NSAIDs have on bone formation in animals is well documented as described above and in Table 1. However, far less is known about NSAID effects on bone formation in humans. In one area, extensive research has shown that NSAID therapy can inhibit bone formation to reduce the severity or incidence of heterotopic bone formation following hip and femoral neck fractures, after hip arthroplasty, or after certain central nervous system injuries that are often associated with heterotopic ossification in humans [80,81,82,83]. Indeed, NSAIDs are now routinely prescribed for patients following hip athroplasty, and acetabular fractures. The mechanism underlying the NSAID-induced reduction in heterotopic bone formation remains unknown.

Currently, we are unaware of any large, prospective, randomized trials to assess the effects of NSAID therapy on bone fracture healing in humans. A few small studies have examined NSAID use and bone healing success in humans (see Table 2). 

**Table 2:** NSAID effects on bone healing: human studies.

Procedure or Injury	Number of Patients	Mean or Median age (yrs)	Follow-up (months)	Drug	Dose(s)	Results	Comments	Ref.
Fracture, Ankle-joint fracture dislocation	Exp:1	Exp:64	10 weeks	Indomethacin	25 mg, QID for 9 weeks	Inhibitory unhealed at 9 weeks		[84]
Fracture, colles	Exp: 48 Con:50	Exp: 62.9 Con: 58.7	12	Flurbiprofen	50 mg, 3–6 times a day for 14 days	Exp: 50% excellent functional result Con: 94% excellent functional result		[86]
Fracture, colles	Exp:21 Con:21	63	12 weeks	Piroxicam	20 mg/day for 8 weeks	Exp: 28% needed surgery Con: none needed surgery		[85]
Acetabular fracture	Exp: 41 Con:34	Exp: 43 Con:47	(Range) Exp:12 Con:11.7	Indomethacin	25 mg, 3x/day for 6 weeks	No difference between grade distribution	Prospective study	[82]
Acetabular fracture	Exp: 72 Con: 16	Exp: 41 Con:	Exp: 13 Con:	Indomethacin	25 mg, QID for 6 weeks	Exp: 11.1% grade iii or iv ho Con: 37.5% grade iii or iv ho	Control patient group is small, study was designed to compare radiation *vs.* NSAID therapy for reduction of ho	[83]
Acetabular fracture	Exp: 74 Con:36	Exp: 39.5 Con: 38.6	3–11	Indomethacin	25 mg, QID for 6 weeks	Inhibitory Exp: 29% non-union Con: 7% non-union	Also compared radiation	[88]
Fracture	Exp: 893 Con: 5781	Exp: 74 Con:74	24	NSAIDs	5–7 times a week	No protective effect on subsequent risk of fractures	BMD & fracture risk assessment	[90]
Fracture	Exp: 32 Con:67	Exp: 35 Con: 38	7+	Ibuprofen or diclofenac	Varied, 1–21 weeks	Inhibitory Exp: 10.74 odds ratio	Retrospective study	[87]
Fracture	Exp1: 214,577 Exp2: 286,850 Con: 214,577	Exp1: 55 Exp2:44 Con:55	Exp1: 3.4 yrs Exp2: .7 years Con: 2.3 yrs	NSAIDs	Exp1: 3+ times per week Exp1: 1–2 times per week	Exp1: 1.47 Exp1 *vs.* Exp2: 1.04	Retrospective study, fracture risk is similar between regular and incidental NSAID users	[91]
Fracture, humeral shaft	Exp: 1,032 Con:8,963	Exp: 78 Con:77	3	Traditional NSAID	Varied, at least 10 days of treatment in a 30 day period	Exposure to NSAID was associated with nonunion	Retrospective study	[89]
Acetabular fracture	Exp: 18 Con: 26	Exp: 41 Con: 37	12+	Indomethacin	25 mg QID for 6 weeks	Exp: 1/18 grade ii ho, 0/18 grade iii or iv Con: 3/26 grade ii ho, 10/26 grade iii or iv ho	Heterotrophic bone ossification study	[80]
Spinal fusion	Exp: 167 Con: 121	Exp: 43 Con: 45	24+	Ketorolac	60 mg IM, then 30 mg/IM very 6–8 h as needed	Inhibitory	Retrospective study; 4.25-fold increase in non-unions	[92]
						Exp: 17% non-union Con: 4% non-union		

* exp = experimental; con = control.

One study conducted by Adolphson *et al.* found that 28% of Colle’s fracture patients who were treated by closed reduction and casting and were treated with piroxicam lost fracture reduction which necessitated subsequent surgical correction. In contrast, all of the Colle’s fracture patients that were treated by closed reduction and casting but with no NSAID therapy healed without surgical intervention [85]. In a double-blind prospective study, flurbiprofen was used in the management of 100 Colles’ fractures [86]. After one year, patients with minimally displaced fractures treated with placebo had significantly better (94%) functional recovery when compared with those treated with flurbiprofen (50%). In contrast, no difference was found between the placebo and flurbiprofen treatment subgroups in the displaced Colles’ fracture group that were treated by surgical fracture reduction after one year.

Retrospective studies indicate that NSAID therapy can significantly impair fracture healing in humans. In a retrospective study designed to assess the effects of reaming on femur fracture healing outcomes, Giannoudis found that the most significant variable affecting femur fracture healing success was NSAID use [87]. The records of 377 femur fracture patients were examined and 32 were found that had developed non-unions. Of the remaining patients, 67 had comparable injuries, co-morbidities, and treatment histories but in these patients, the femur fractures had healed. Of the 32 non-union patients 62.5% had used NSAIDs while only 13.4% of patients with healed femur fractures had used NSAIDs. A compelling retrospective study was performed by Burd *et al.* [83]. Previously, this group had performed a prospective, randomized trial to compare the efficacy of localized radiation therapy *versus* systemic indomethacin therapy to reduce the incidence or severity of heterotopic ossification following acetabular fractures [83]. Using this same of set of patients, Burd *et al.* queried whether the indomethacin therapy affected the healing of any additional long bone fractures in the study patients [88]. They found a non-union rate of 7% in the localized radiation therapy group and a 26% non-union rate in the indomethacin therapy group. One retrospective study, however, failed to identify any negative effect of NSAID use on humerus fracture healing [88]. Approximately 10% of 9,995 humeral shaft fracture patients in the study used NSAIDs sometime in the 90 days after fracture. Only 105 of 9995 patients had a subsequent surgery to treat a non-union and 33 of the 105 non-union patients had used NSAIDs. Other studies also indicate that NSAID use can increase fracture risk and decrease bone mineral density [90,91]. We are unaware of any human studies that have specifically assessed the effects of COX-2 selective inhibitors on fracture healing.

The negative effects of NSAID therapy on spinal fusion success in humans has been documented in a retrospective study. Post-operative ketorolac use significantly impaired spinal fusion success [92]. No study examining COX-2 selective NSAID use and spinal fusion success in humans has been reported besides the aforementioned ones published and subsequent retracted by Reuben. Since ketorolac is a non-specific NSAID, no conclusion can be made as to whether the COX-2 selective NSAIDs will have similar negative effects on spinal fusion success. Recent experiments in rabbits suggest that celecoxib may not have a negative effect on spinal fusion success [41].

## 6. Role of COX-2 during Bone Healing

Clearly, COX-2 is a positive regulator of fracture healing. However, the mechanisms by which COX-2 promotes healing or by which COX-2 inhibition impairs bone healing are unknown. It is likely that the loss of COX-2 function with NSAID use alters many cellular pathways required for bone healing and therefore multiple molecular pathways are involved.

One theory is that COX-2 function is essential for mesenchymal cell differentiation into osteoblasts, which is necessary for normal fracture healing. Therefore, inhibition of COX-2 activity is expected to negatively impact osteogenesis. Zhang *et al.* found that bone marrow cell cultures from COX-2 knockout mice produced less osteoblasts than wild-type mice but that treatment with BMP-2 and prostaglandin E_2_ could reverse this effect [93]. Chikazu *et al.* also demonstrated that COX-2 contributes to BMP-2 induced osteoblastic differentiation *in vitro* and *in vivo* during ectopic bone formation [94]. However, COX-2 null mice have normally formed skeletons and satisfactory post-natal growth indicating that loss of COX-2 activity does not inherently prevent stem cells from differentiating into osteoblasts.

Another theory is that the pain relief afforded by NSAID treatment enables experimental animals to weight bear on the injured limb too early or often, which leads to re-injury and delayed healing. This theory was tested in the rat femur fracture healing model using celecoxib (3 mg/kg/day or 6 mg/kg/day) and acetaminophen (60 mg/kg/day or 300 mg/kg/day) to provide pain relief for 10 days post-fracture [73]. Results from radiographs and mechanical testing demonstrated that celecoxib inhibited healing while acetaminophen did not. In a subsequent study, pain relief from femur fractures in rats treated with acetaminophen, celecoxib (10 mg/kg, BID), and SCIO-469, a p38 kinase-α inhibitor treatment was measured [95]. All the drugs provided significant pain relief. However, only celecoxib treatment was associated with impaired healing in previous studies. These data suggest that analgesia only is not sufficient to account for the negative effects of celecoxib or NSAID treatment on fracture healing. Thus the theory that analgesia induced re-injury causes the delay in healing is not supported by the current experimental evidence.

A third theory is that COX-2 dependent prostaglandins promote angiogenesis that is required for fracture healing [96,97]. Studies have shown that prostaglandins and COX-2 regulate pro-angiogenic microenvironments. These microenvironments are vital to cellular signaling responses and may be important for bone regenerative cells and tissues [98]. Murnaghan *et al.* supported this theory by showing that rofecoxib treatment reduced blood flow across the fracture gap while impairing fracture healing in a mouse fracture model [75]. Conversely, Xie *et al.* demonstrated that treatment of COX-2 null mice with a prostaglandin E_2_ receptor 4 (EP4) agonist increased callus vascularity and restored fracture healing [99]. These data indicate that COX-2 has a role in promoting angiogenesis during fracture healing. 

A fourth theory is that loss or inhibition of COX-2 shunts arachidonic acid into the 5-lipoxygenase (5-LO) pathway which alters the inflammation response and negatively affects bone healing [99]. 5-LO is the key enzyme in the production of leukotrienes from arachidonic acid and inhibition of 5-LO accelerates fracture healing in rats [100]. Leukotrienes can decrease osteoblast proliferation and activity *in vitro* while stimulating osteoclast formation and activity [101,102,103,104,105,106]. Fracture callus leukotriene B_4_ levels are 4.4-fold higher in COX-2 null mice, consistent with an arachidonic acid shunting mechanism (Manigrasso and O’Connor, submitted). Together, these data indicate that 5-LO activity normally acts to inhibit fracture healing and because loss of COX-2 increases 5-LO activity, healing is impaired.

In the rat closed femur fracture model, celecoxib treatment significantly affects healing in multiple ways [100]. Celecoxib treatment reduced callus cell proliferation rates without altering callus cell numbers, suggesting that cell migration to the fracture site is not affected. Celecoxib treatments also lead to increases in aggrecan and Type II collagen mRNA levels which are indicative of chondrocyte differentiation and cartilage formation. This is consistent with histological and histomorphometric measurements showing formation of a cartilaginous callus in COX-2 deficient animal models. However, Type X collagen mRNA levels were significantly reduced relative to the high the Type II collagen and aggrecan mRNA levels, indicating that the callus chondrocytes in the celecoxib treated rats failed to progress into hypertrophy. This would in turn reduce formation of calcified cartilage, impair endochondral ossification, and prevent healing.

Though there are many theories regarding the role of COX-2 in fracture healing, many basic questions are still unanswered. For instance, we do not know when or in which cells COX-2 is expressed during fracture healing. Nor do we know the repertoire of prostaglandins and other eicosanoids produced during fracture healing or how loss of function in one of the arachidonic acid metabolizing enzymes affects the activity of the remaining enzymes. Understanding these and other basic parameters of arachidonic acid metabolism during fracture healing will provide critical information needed to refine testable hypotheses for elucidating the function of COX-2 during bone regeneration. 

## 7. Summary　

This review strived to provide an unbiased analysis of the current literature in regard to non-steroidal anti-inflammatory drugs and their effect on bone healing. Overwhelmingly, this review demonstrates that NSAIDs inhibit or delay fracture healing to a greater or lesser degree, depending on the specific drug, its preparation, mode of administration, and ability to inhibit COX-2. The majority of animal studies and the few human studies that exist support this conclusion. However, further research to determine if the analgesic effects of NSAIDs are beneficial in treating post-traumatic and postoperative wound edema. Since it is increasingly clear that the function of COX-2 is critical for bone regeneration, defining the risk-benefit ratio for NSAID use is critical. Patient co-morbid conditions, such as, fracture severity, advanced age, diabetes, and cardiovascular health, will need to be considered when assessing the risk-benefit ratio. Future research is needed to define the role of COX-2 in bone regeneration and whether NSAID therapy will further impair fracture healing or other regenerative processes in the presence of these co-morbid conditions. 
